# Taxonomic and metabolic development of the human gut microbiome across life stages: a worldwide metagenomic investigation

**DOI:** 10.1128/msystems.01294-23

**Published:** 2024-03-05

**Authors:** Leonardo Mancabelli, Christian Milani, Rosita De Biase, Fabiana Bocchio, Federico Fontana, Gabriele Andrea Lugli, Giulia Alessandri, Chiara Tarracchini, Alice Viappiani, Flora De Conto, Antonio Nouvenne, Andrea Ticinesi, Ovidio Bussolati, Tiziana Meschi, Rossana Cecchi, Francesca Turroni, Marco Ventura

**Affiliations:** 1 Department of Medicine and Surgery, University of Parma, Parma, Italy; 2 Interdepartmental Research Centre "Microbiome Research Hub", University of Parma, Parma, Italy; 3 Laboratory of Probiogenomics, Department of Chemistry, Life Sciences and Environmental Sustainability, University of Parma, Parma, Italy; 4 GenProbio srl, Parma, Italy; 5 Parma University Hospital, Parma, Italy; Medical Research Council Toxicology Unit, United Kingdom

**Keywords:** human gut microbiome, human life span, shotgun metagenomics, human microbiota

## Abstract

The human gut microbiota is a dynamic community of microorganisms that undergo variable changes over the entire life span. To thoroughly investigate the possible fluctuations of the microbiota throughout human life, we performed a pooled analysis of healthy fecal samples across different age groups covering the entire human life span. Our study integrated data from 79 publicly available studies and new stool samples from an Italian cohort, i.e., the Parma Microbiota project, resulting in 6,653 samples processed through the shotgun metagenomic approach. This approach has allowed species-level taxonomic reconstruction of the gut microbiota and investigation of its metabolic potential across the human life span. From a taxonomic point of view, our findings confirmed and detailed at species-level accuracy that the microbial richness of the gut microbiota gradually increases in the first stage of life, becoming relatively stable during adolescence. Moreover, the analysis identified the potential core microbiota representative of distinct age groups, revealing age-related bacterial patterns and the continuous rearrangement of the microbiota in terms of relative abundances across the life span rather than the acquisition and loss of taxa. Furthermore, the shotgun approach provided insights into the functional contribution of the human gut microbiome. The metagenomic analysis revealed functional age-related differences, particularly in carbohydrate and fiber metabolism, suggesting a co-evolution of the microbiome assembly with diet. Additionally, we identified correlations between vitamin synthesis, such as thiamine and niacin, and early life, suggesting a potential role of the microbiome in human physiology, in particular in the functions of the host’s nervous and immune systems.

**IMPORTANCE:**

In this study, we provided comprehensive insights into the dynamic nature of the human gut microbiota across the human life span. In detail, we analyzed a large data set based on a shotgun metagenomic approach, combining public data sets and new samples from the Parma Microbiota project and obtaining a detailed overview of the possible relationship between gut microbiota development and aging. Our findings confirmed the main stages in microbial richness development and revealed specific core microbiota associated with different age stages. Moreover, the shotgun metagenomic approach allowed the disentangling of the functional changes in the microbiome across the human life span, particularly in diet-related metabolism, which is probably correlated to bacterial co-evolution with dietary habits. Notably, our study also uncovered positive correlations with vitamin synthesis in early life, suggesting a possible impact of the microbiota on human physiology.

## INTRODUCTION

The human gut microbiota, a rich and variable consortium of microorganisms residing in the intestinal tract (1–3), plays a crucial role in numerous aspects of human biology, including metabolic health to immune functions (3–5). This complex community, predominantly bacterial, undergoes dynamic changes throughout an individual’s life, reflecting the interplay between microbial communities and host development (6, 7). In early life, the gut is gradually colonized, with its microbiota being highly dynamic and influenced by factors such as delivery mode and breastfeeding (8–10). Initially, it is characterized by Actinomycetota and Pseudomonadota (formerly known as Actinobacteria and Proteobacteria, respectively) (2, 11, 12). As children grow, significant shifts occur in the gut microbiota (13, 14), adapting to dietary changes from breastfeeding to solid foods (15).

Despite the current limited research focusing on the gut microbiota during human adolescence, it is widely suggested that, during this crucial window of time, the gut microbial community undergoes significant transformations and evolutions (16, 17). In fact, hormonal changes, dietary preferences, and lifestyle factors may exert influences on the gut microbial ecosystem (18–21). Notably, during adolescence, the microbiota tends to shift toward that of an adult, which is characterized by an increase in microbial genera belonging to the phyla Bacteroidota and Bacillota (formerly known as Bacteroidetes and Firmicutes, respectively), such as *Bacteroides*, *Prevotella*, *Blautia*, and *Faecalibacterium* (22).

Compared with infants and adolescents, adults are endowed with a microbiota characterized by increased stability and resilience (23–25). Its composition is strongly influenced by lifestyle-related factors such as diet (20, 26) and physical activity (27, 28). Numerous studies have revealed that a diverse and stable gut microbiota in adults is associated with improved metabolic and immune health, while an imbalance in composition can be linked to various pathological conditions (1, 29, 30). In fact, the balance between the major phyla and genera of the adult gut microbiota plays a crucial role in ensuring the host’s well-being and fulfilling essential metabolic and immunological functions (1, 24).

In the later stages of life, elderly people experience another shift in their gut microbiota composition, which is characterized by a decrease in bacterial richness, particularly in beneficial species such as *Bifidobacterium*, *Akkermansia*, and members of *Clostridium* Clusters IV (31, 32). These changes may affect immune function, nutrient absorption, and susceptibility to age-related conditions, such as frailty and chronic diseases (31, 33, 34).

In this context, comprehensive studies with robust statistical power that investigate the detailed evolution of the gut microbiota across the life span have been notably limited and mainly focused on specific age groups (11, 35–39). Furthermore, a critical knowledge gap remains despite the wealth of knowledge gained in recent years. Currently, most of the existing studies are based on the 16S rRNA gene profiling approach (40), providing insights of the microbiota composition at the genus level but lacking the precision required for species-level analysis (41).

Given these critical gaps of knowledge, our study aims to perform the most extensive gut microbiota analysis based on publicly available shotgun metagenomic data sets regarding studies of the healthy human microbiome across life spans. In detail, we have assembled a very complete data set encompassing 6,653 samples representing various age groups and geographical regions, including 467 samples that originated from a still ongoing local population study, the Parma Microbiota project. This approach enables a more in-depth examination of taxonomic compositions at the species level and the microbiome’s functionality, providing a comprehensive and statistically robust exploration of the intricate relationship between age and microbiome composition.

## RESULTS

### Data set selection

An extensive metagenomic data set search was performed to retrieve the largest number of publicly available shotgun metagenomic studies related to the human microbiome of healthy individuals. In detail, we collected data from 79 publicly available data sets that included healthy human fecal samples based on Illumina shotgun metagenomic methodologies (Fig. S1a; Table S1). In detail, in this pooled analysis, we included only studies in which it was possible to clearly identify the healthy status and the age of the individuals through the reported metadata. Moreover, as part of the Parma Microbiota project, fresh fecal samples from 467 healthy Italian individuals were collected, sequenced, and analyzed (Table S1). Thus, the pooled analysis included a total of 6,653 healthy fecal samples ranging from birth to over 100 years old (Tables S1 and S2) with a robust statistical representation of all the different age groups (see below).

### Intra-individual variability across human life

The 6,653 stool samples collected in this pooled analysis were used to assess the microbiota composition through the METAnnotatorX2 software (42) following the standard filtering parameters reported in the manual with *Homo sapiens* reads removal. The downloaded and the sequenced fastq files were processed with the same bioinformatic pipeline to prevent biases, resulting in a total of 110,384,672,113 reads with an average per sample of 14,885,023 ± 16,560,451 after quality and human sequence filtering (Table S2). To optimize the taxonomical analysis, following a shallow shotgun metagenomic approach (42, 43), we decided to analyze, after quality and human sequence filtering, a random subset of up to 100,000 reads for each sample, obtaining a total of 424,267,507 classified reads with an average per sample of 63,771 ± 13,611 (Table S2). As previously reported, this approach allowed the optimization of the bioinformatic pipeline, ensuring accurate taxonomic profiling. Additionally, it promotes reliable profile comparisons by mitigating disparities resulting from variations in the total number of analyzed reads (42, 43).

**Fig 1:**
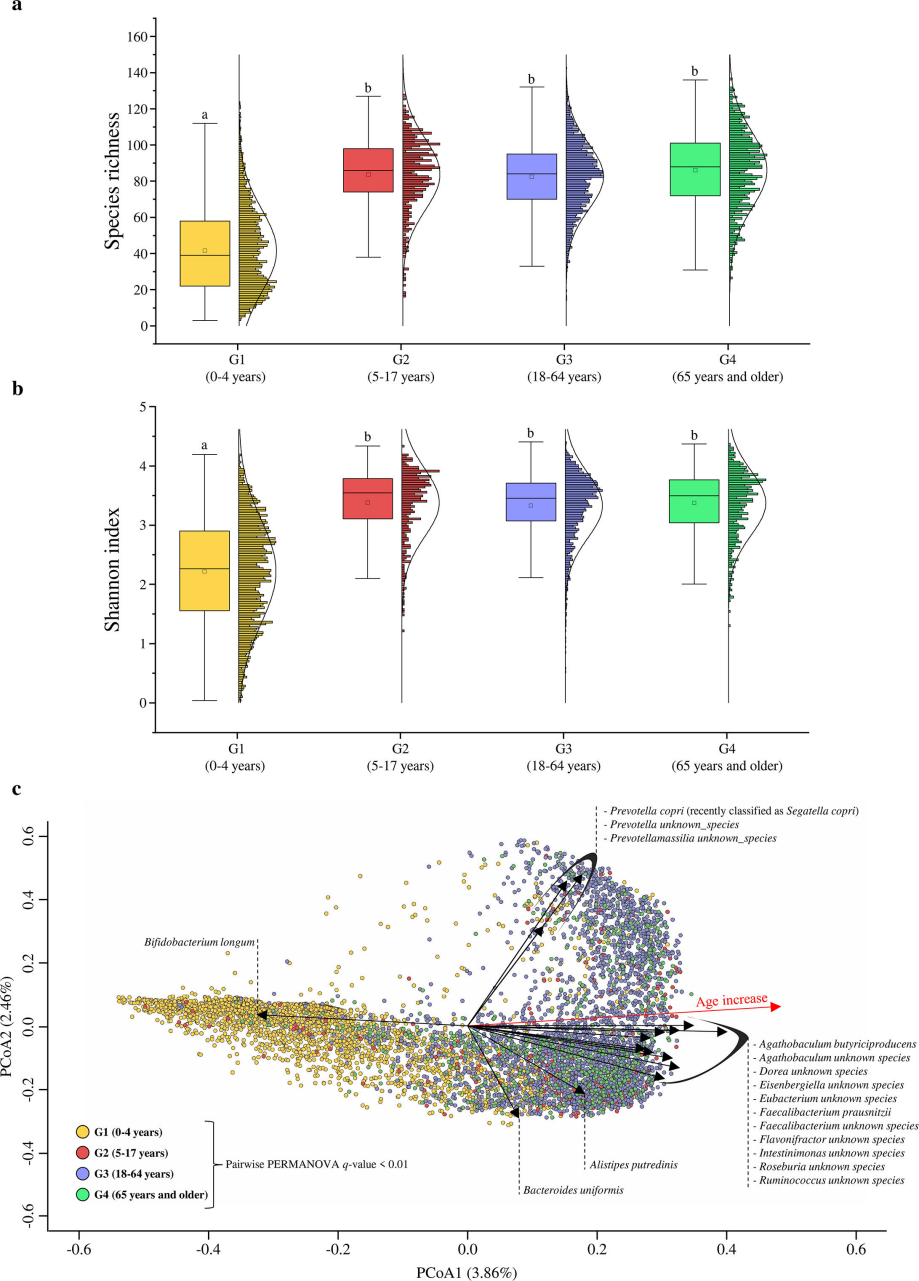
Evaluation of microbial biodiversity. Panel (a**) **displays the whisker plot representing the species richness identified by subjects of each age group. The *x*‐axis represents the different age groups, while the *y*‐axis indicates the number of species. The 25th and 75th percentiles determine the boxes. The whiskers are determined by the 1.5 interquartile range (IQR). The line in the boxes represents the median, while the square represents the average. Different lowercase letters indicate significant differences at *P* <  0.05 calculated through pairwise Kruskal-Wallis test analyses. In detail, groups with the same letter are not significantly different from each other, while groups with different letters are considered statistically distinct. Panel (b**) **reports the whisker plot representing the alpha diversity calculated through the Shannon index identified by subjects of each age group. The *x*‐axis represents the different age groups, while the *y*‐axis indicates the Shannon index. The 25th and 75th percentiles determine the boxes. The whiskers are determined by the 1.5 IQR. The line in the boxes represents the median, while the square represents the average. Different lowercase letters indicate significant differences at *P* <  0.05 calculated through pairwise Kruskal-Wallis test analyses. In detail, groups with the same letter are not significantly different from each other, while groups with different letters are considered statistically distinct. Panel (c**) **shows the pooled analysis of PCoA, subdivided by age groups. The black rows indicate the bacterial species with significant fittings (envit fit *P* < 0.005).

The results generated using METAnnotatorX2 software (42) were employed to assess the biodiversity of each sample. In detail, to explore potential variations in species richness throughout the human life span, we categorized the samples into four age groups, that is, G1 (0–4 years), G2 (5–17 years), G3 (18–64 years), and G4 (65 years and older) (Fig. S1), following the guidelines provided by the World Health Organization (WHO) (44). The analysis revealed an increase in bacterial species abundance with age, as highlighted by a pairwise Kruskal-Wallis test (*P* < 0.01). Specifically, there was a substantial difference between G1 (average of 42 ± 23) and G2, G3, and G4 groups (average of 84 ± 20, 83 ± 20, and 86 ± 21, respectively) (Fig. 1A). No significant differences were identified between G2, G3, and G4 groups (Fig. 1A). These results support the notion that the human gut microbiome undergoes developmental changes in the early stages of life, showing increasing complexity in terms of bacterial species until the human host reaches childhood and adolescence (5–17 years) (23). Subsequently, in the later stages of life, the gut microbiome reaches stability, with the number of bacterial species remaining relatively constant. In addition, a subdivision of the samples into further age subgroups (Fig. S1a) showed no significant differences between G1a (0–1 month) and G1b (1–6 months), indicating the presence of a heterogeneous and complexity-varying microbiota (14). Moreover, the G1c (6 months–1 year) and G1d (1–4 years) groups showed a significant trend of increase in species number until reaching the G2a group (5–10 years), where the gut microbiota appears to achieve a stable state that persists in subsequent age groups (pairwise Kruskal-Wallis test *P* > 0.05) (Fig. S1a).

### Inter-individual variability between different stages of life

Principal coordinate analysis (PCoA), based on the Bray-Curtis dissimilarity matrix, was used to evaluate the inter-individual differences between age groups. In detail, the statistical analysis based on PERMANOVA (Table S3) revealed a clear division between the groups (pairwise PERMANOVA *q* < 0.01). Furthermore, the pairwise pseudo-*F* values (Table S2) evaluation highlighted a clear separation between the G1 group and the others, which exhibited heterogeneity among themselves (Fig. 1B). This division suggested a distinct microbiota composition of the G1 group samples, indicating a significant relationship between age and microbiota structure. Further fitting analyses, considering age and bacterial species as variables, identified *Bifidobacterium longum* as a key microbial taxon (envit fit *P* = 0.002, *r*² = 0.2751). This species appeared to exhibit a significant negative relationship with increasing age and is strongly associated with the G1 group. In contrast, other bacterial species with significant fittings (envit fit *P* < 0.005) displayed a positive relationship with increasing age, forming three distinct clusters. A prevalence of species from the *Prevotella* genus, such as *Prevotella copri* (recently classified as *Segatella copri*), characterized the first cluster. *Bacteroides uniformis* and *Alistipes putredinis* characterized the second cluster, while the third cluster exhibited diverse bacterial genera commonly associated with the adult gut microbiota, including *Ruminococcus*, *Roseburia*, and *Faecalibacterium*. These three clusters appeared to reflect the typical enterotypes of adults (45) but provided greater species-level detail. Moreover, the fitting analysis could highlight that enterotype 3, which is associated with the genus *Ruminococcus*, might instead consist of a complex community of bacteria with a less clear dominance of driver species (46, 47).

In addition to the age parameters, we also tested the potential impact of the variables related to the BioProject and nation of the samples on microbiota composition using PERMANOVA analysis. This analysis revealed *q* < 0.01 for both parameters, indicating statistical significance. However, the effect sizes measured by pseudo-*F* values were 16.7 and 11.4 for BioProject and geographical origin, respectively. These findings suggest that, while these two parameters may have a statistically significant impact on microbiota composition, their effect appears to be relatively modest. This observation could be assigned, in part, to the heterogeneous nature of the data set.

### Identification of possible specific bacterial patterns related to different human stages of life

The METAnnotatorX2 software (42) allowed to obtain a detailed taxonomical profile at the species level for each sample. In detail, the sample size employed in this pooled analysis allowed the identification of age-specific core microbiota within each age group (Table S4). Core microbiota members were defined with a prerequisite of a minimum prevalence of 50% and an average relative abundance above 0.1% per age group. These criteria were chosen following the main standards in the field (48) and considering taxonomic complexity at the species level. Additionally, species with a prevalence between 30% and 50%, along with an average relative abundance above 0.1% were classified as accessory taxa. The core microbiota of each age group highlighted that G1 group displayed the simplest core composition, including seven species mainly represented by *B. longum* and *Escherichia coli* species (Table S4). In contrast, G2, G3, and G4 groups displayed larger and more diversified core microbiota, composed of 68, 57, and 63 species, respectively (Table S4). Accessory taxa exhibited a similar upward trend to the core, further validating the hypothesis that the gut microbiota demonstrates substantial compositional variability during the early developmental stages and then shifts to taxonomic stability with advancing age (2, 23).

In order to determine the mainly representative species that compose the human gut microbiota across the life span, the bacterial species common to both the core and accessory microbiota across all age groups were selected (Table 1). This extensive screening yielded 29 bacterial species, including *B. uniformis*, which was the only species present in all cores, and *Bacteroides fragilis* that was the only ubiquitous species as an accessory taxa (Table 1). Notably, 21 taxa were identified as accessory species exclusively in the G1 group, subsequently transitioning to become core members in the G2, G3, and G4 groups. This pattern suggests that the development of the microbiota is characterized not only by the acquisition and loss of taxa but also by a significant rearrangement in their relative abundances. The early acquisition of certain species, pivotal in later life stages, highlights the critical role of microbiota enrichment during the initial phases of development. Moreover, among the microbial taxa representing the core microbiota of the G1 age group, only *B. longum* remains highly prevalent and abundant even in the G2 age group and then decreases in the subsequent G3 and G4 age groups, constituting one of the accessory taxa. These results suggested that these taxa persisted across the life span but exhibited dynamic interactions, shifting their prevalence over time. This dynamic co-existence could imply the formation of a complex ecosystem that could potentially reach a climax condition (23).

**TABLE 1:** Mainly representative species that compose the human gut microbiota across the life span^*a*^

	Prevalence >50% and relative average abundance >0.1%				Relative average abundance			
Taxonomy	G1 (*N* = 3,100)	G2 (*N* = 366)	G3 (*N* = 2,632)	G4 (*N* = 555)	G1 (*N* = 3,100)	G2 (*N* = 366)	G3 (*N* = 2,632)	G4 (*N* = 555)
*B. longum*	**69.71%**	**75.68%**	36.17%	41.44%	9.03%	2.53%	0.55%	1.52%
*E. coli*	**64.84%**	43.44%	**58.51%**	**54.77%**	6.69%	0.87%	1.33%	2.26%
*Bacteroides* unknown_species	**56.97%**	**96.72%**	**92.90%**	**88.47%**	0.66%	1.23%	1.23%	1.19%
*Clostridium* unknown_species	**56.84%**	**96.45%**	**96.12%**	**95.86%**	0.48%	0.53%	0.60%	0.58%
*Blautia* unknown_species	**53.42%**	**98.09%**	**98.52%**	**98.38%**	0.58%	1.57%	2.08%	1.57%
*B. uniformis*	**51.81%**	**94.54%**	**89.40%**	**88.29%**	2.99%	4.10%	3.40%	4.10%
Ruminococcus unknown_species	**50.58%**	**97.81%**	**97.26%**	**97.48%**	0.45%	1.54%	1.68%	1.67%
*Blautia wexlerae*	48.39%	**92.62%**	**87.42%**	**84.50%**	1.82%	1.77%	1.68%	1.45%
*Bifidobacterium* unknown_species	46.87%	**60.93%**	31.08%	42.16%	0.58%	0.29%	0.11%	0.19%
*Flavonifractor plautii*	46.87%	**83.33%**	**66.53%**	**78.38%**	0.57%	0.37%	0.29%	0.40%
*Phocaeicola vulgatus*	42.06%	**84.43%**	**72.49%**	**73.69%**	1.31%	1.44%	1.15%	1.12%
*Bacteroides thetaiotaomicron*	40.90%	**84.70%**	**72.72%**	**73.87%**	0.82%	0.78%	0.59%	0.74%
*Phocaeicola dorei*	39.45%	**80.60%**	**63.11%**	**66.13%**	2.05%	1.51%	1.29%	1.37%
*Eubacterium* unknown_species	38.71%	**94.54%**	**95.90%**	**95.86%**	0.26%	0.99%	1.48%	1.29%
*Parabacteroides distasonis*	37.84%	**82.51%**	**69.38%**	**77.48%**	1.67%	1.15%	0.87%	1.07%
*Enterocloster* unknown_species	37.23%	**87.98%**	**90.35%**	**89.55%**	0.12%	0.16%	0.22%	0.21%
*Roseburia* unknown_species	36.74%	**93.72%**	**95.59%**	**94.05%**	0.22%	0.59%	1.14%	0.84%
*Faecalibacterium* unknown_species	36.65%	**93.44%**	**96.01%**	**94.77%**	1.17%	4.11%	3.23%	3.39%
*Faecalibacterium prausnitzii*	36.35%	**92.62%**	**96.05%**	**93.69%**	0.97%	3.41%	2.94%	3.12%
*Bacteroides xylanisolvens*	35.84%	**79.78%**	**56.34%**	**64.68%**	0.52%	0.63%	0.56%	0.55%
*Phocaeicola* unknown_species	35.68%	**79.23%**	**79.71%**	**74.59%**	0.21%	0.48%	0.65%	0.53%
*Anaerostipes hadrus*	34.35%	**82.51%**	**63.87%**	**58.38%**	1.11%	1.23%	1.09%	1.09%
*B. fragilis*	33.58%	43.44%	31.50%	37.48%	2.99%	1.25%	0.43%	0.78%
*Coprococcus* unknown_species	33.55%	**87.43%**	**88.87%**	**86.31%**	0.14%	0.48%	0.75%	0.58%
*Agathobacter rectalis* (formerly known as *Eubacterium rectale*)	31.29%	**90.71%**	**92.86%**	**87.39%**	0.99%	3.23%	4.11%	2.70%
*Blautia massiliensis*	31.03%	**84.70%**	**75.68%**	**72.43%**	0.41%	1.01%	0.77%	0.62%
*Dorea* unknown_species	30.77%	**89.89%**	**89.02%**	**85.95%**	0.12%	0.39%	0.58%	0.41%
*Bacteroides ovatus*	30.35%	**68.58%**	**51.41%**	**51.53%**	0.44%	0.33%	0.22%	0.23%
*Roseburia intestinalis*	30.06%	**80.60%**	**84.38%**	**81.26%**	0.74%	0.65%	1.01%	0.76%

^*a*^ In detail, only the bacterial species common to both the core and accessory microbiota across all age groups were reported. The bacterial taxa belonging to the core microbiota were highlighted in bold.

**Fig 2:**
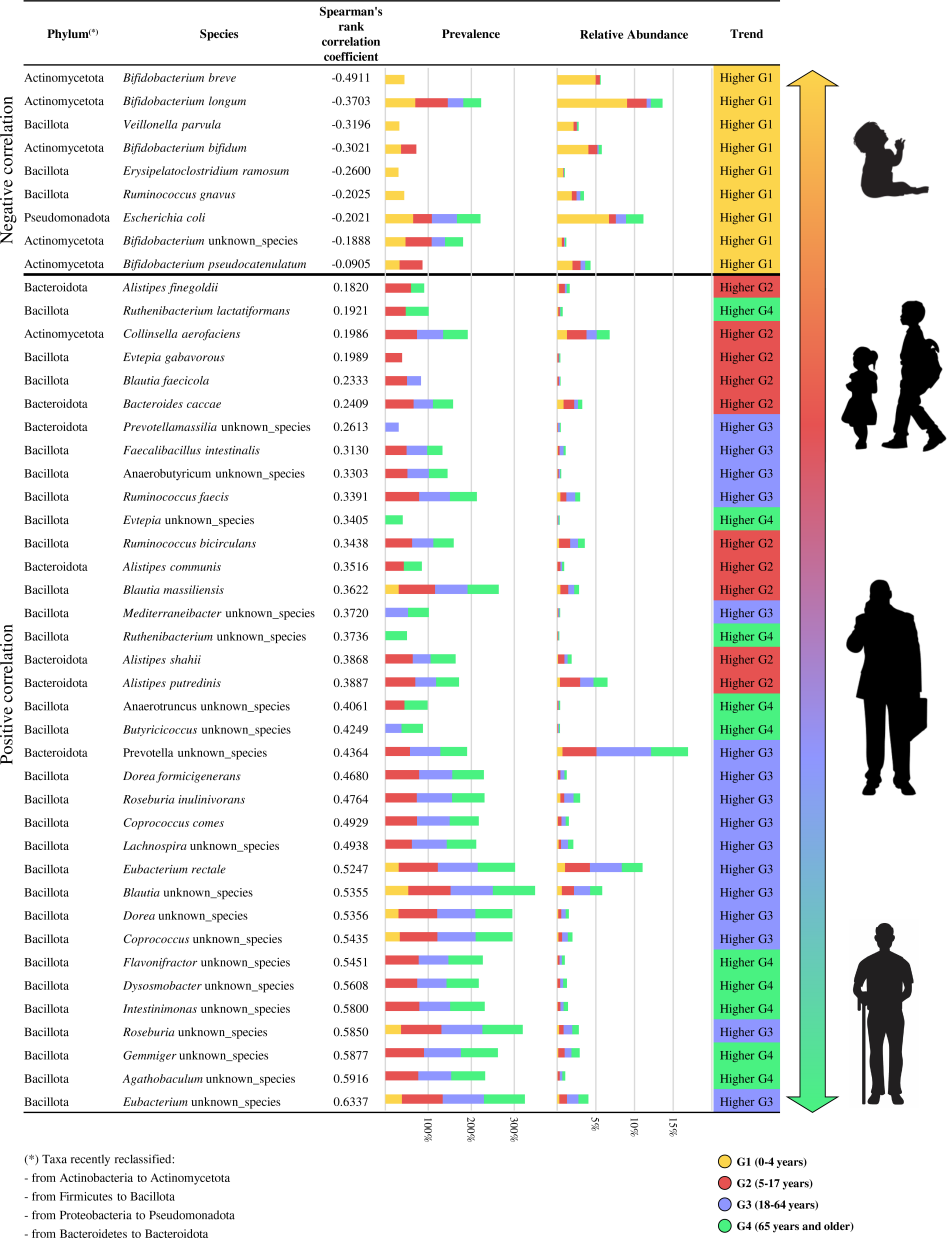
Correlation analysis between the bacterial species and the age of the individuals included in pooled analysis. In detail, only the bacterial taxa that showed a significant Spearman’s rank correlation coefficient and significantly higher relative abundance in at least one of the age groups calculated through ANOVA test analysis and multiple comparison analyses Tukey’s HSD test were reported.

In addition, a dedicated correlation analysis was conducted to highlight the bacterial species significantly associated with aging. Based on Spearman’s rank correlation coefficient, the correlation analysis revealed a total of 104 taxa with a significant relationship with age (*P* < 0.01) (Table S5). Among these taxa, we focused on those that showed significantly higher relative abundance in at least one of the age groups (multiple comparison analyses Tukey’s (honestly significant difference) HSD *P* < 0.05, Fig. 2), resulting in a total of 45 taxa. Within this selection, we identified nine taxa with a negative correlation and 36 taxa with a positive correlation to age (Fig. 2). Notably, the nine taxa with a negative correlation were more abundant in the G1 group and were primarily represented by species belonging to the *Bifidobacterium* genus, such as *Bifidobacterium breve*, *B. longum*, *Bifidobacterium bifidum*, and *Bifidobacterium pseudocatenulatum*, along with species characteristic of the infant microbiota, such as *Veillonella parvula*, *Ruminococcus gnavus*, and *E. coli* (11, 12, 38). Notably, the increase of the correlation value showed a trend related to increasing age, except for the characteristic taxa of the G4 group, which corresponds to elderly individuals. These taxa, such as *Ruthenibacterium lactatiformans*, *Anaerotruncus* unknown_species, and *Butyricicoccus* unknown_species, exhibited a more heterogeneous distribution in terms of correlation values despite being more present in the G4 group, suggesting an increase in variability of the intestinal microbiota composition in this age group.

### Exploring microbial functional diversity across the life span

**Fig 3:**
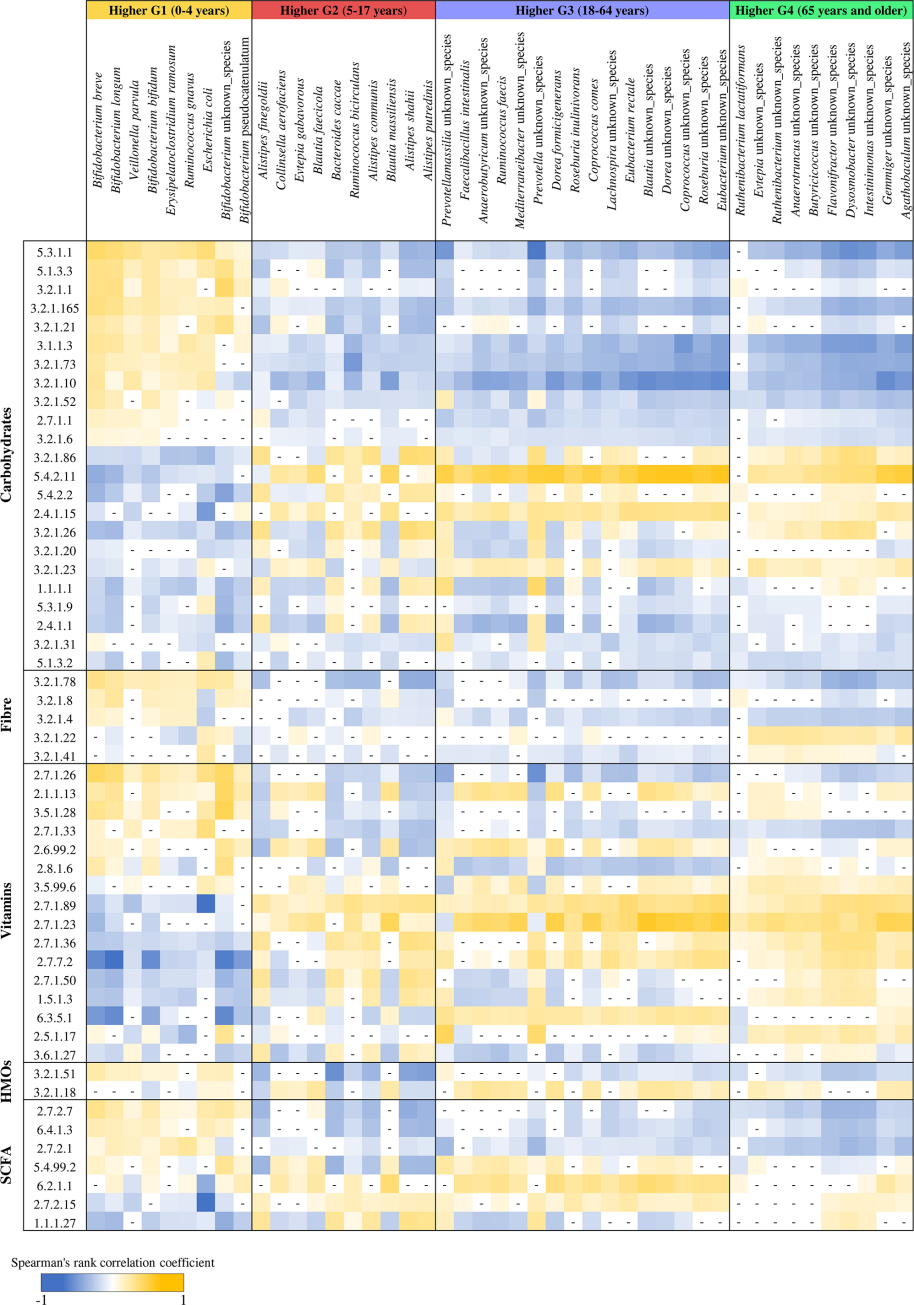
Correlation analysis between the bacterial species and enzymatic reaction identified in pooled analysis. The red color indicated negative correlations, while the green color represented positive correlations. Only the main key enzymes involved in the human diet resulted in statistical significance; Spearman’s rank correlation coefficient were reported.

The taxonomical analysis of the 6,653 healthy fecal samples across the life span revealed possible specific bacterial patterns correlated to the different age groups. In this context, in order to explore the genetic features characterizing each microbiome sample, we performed a screening of the microbially driven metabolic enzymatic reactions based on the MetaCyc database (49) and the Enzyme Commission (EC) classification. In detail, the enzymatic reactions that were revealed through the metagenomic analysis were used to perform a correlation analysis with the 104 bacterial species exhibiting a significant relationship with age (see above). Afterward, focusing on the core and accessory bacterial taxa that were statistically associated with each age group and on the main key enzymes involved in the metabolism of the various components of the human diet or in the metabolism of the main microbial metabolic products important for the host (see more detail in Materials and Methods), the correlation analysis seemed to highlight possible specific correlations between the age groups and the metabolic capability of the gut microbiome (Fig. 3). In detail, the functional analysis revealed a different correlation between the EC involved in the metabolism of carbohydrates and the bacterial species characterizing the four age groups, highlighting a greater similarity between groups G2, G3, and G4 compared with group G1 (Fig. 3). Curiously, EC 3.2.1.23, that is, beta-galactosidase, exhibited a significant positive correlation with G1 group compared with groups G2, G3, and G4. This result confirms the possible association between the composition of the intestinal microbiome and the host’s milk-based diet. Indeed, group G1, which is associated with infants, is likely influenced by a milk-based diet, where beta-galactosidase is essential for breaking down lactose, the prevalent carbohydrate source in milk (50). Similarly, the bacterial taxa representative of the G1 group showed predominantly negative correlations with EC enzymes involved in fiber metabolisms. This result could probably be linked to the infant’s low-fiber diet (51), highlighting the possible relationship between microbiome-EC enzyme and the host’s diet.

Furthermore, the analysis of enzymes related to the biosynthesis of B vitamins also revealed a specific correlation between microbiome composition and human life span. Notably, the G1 group showed a positive correlation with enzymes involved in thiamine, i.e., 2.7.1.89, 2.7.1.50, and 3.6.1.27, and niacin, i.e., 2.7.1.23 and 6.3.5.1, biosynthesis in contrast to the G2, G3, and G4 groups. This observation highlighted the importance and specificity of microbial groups characterizing the G1 group, such as *B. breve*, *B. longum*, *B. bifidum*, *V. parvula*, and *E. coli*, which could contribute to the biosynthesis of these vitamins impacting the physiological development of the host.

### Investigation of the possible impact of geographical origin on the human microbiome across the host life span

**Fig 4:**
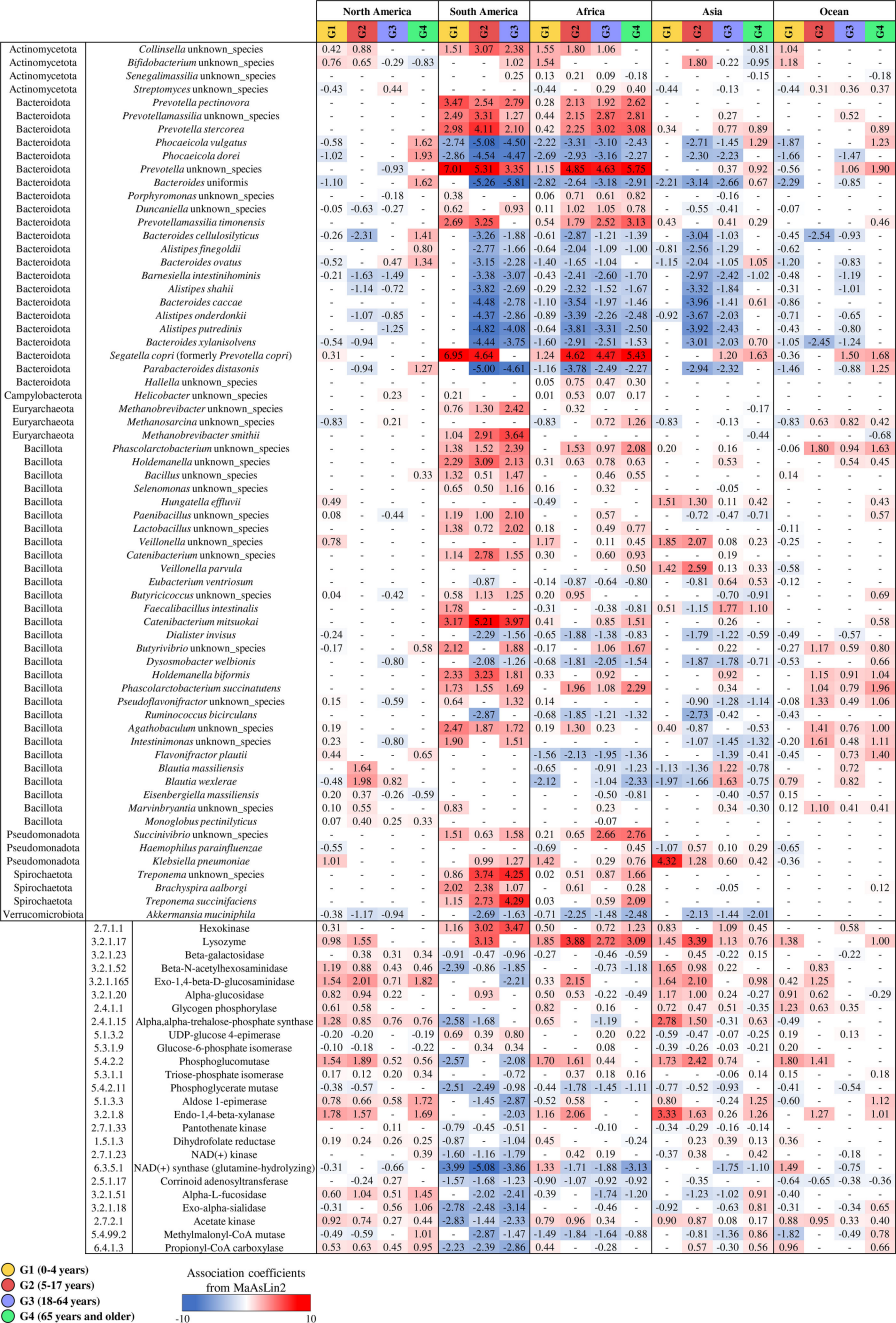
Multivariate analysis through MaAsLin2 software based on bacterial species, age groups, and geographical origin. Significant positive correlations are reported in red, while significant negative correlations are reported in blue.

In order to identify potential variations in the human gut microbiota based on geographical origin, in particular with continent of origin, we performed an exploratory and preliminary multivariable statistical analysis based on MaAsLin2 software using the most representative population, i.e., the European population, as a reference for each pairwise comparison (52) (see more detail in Materials and Methods). The analysis revealed marked and significant differences in the microbiota profiles (Table S6). In detail, individuals from South America and Africa showed more significant differences than those from Europe (Fig. 4), while samples from North America showed the fewest significant differences (Fig. 4). Notably, South America and Africa exhibited a similar taxonomical correlation trend, revealing a positive correlation with species belonging to *Prevotella*, *Prevotellamassilia*, and *Treponema* genera and a negative correlation with bacterial species belonging to the *Phocaeicola*, *Bacteroides*, and *Alistipes* genera. Curiously, these trend correlations appeared consistent across different age groups, indicating a degree of temporal stability in the microbiota composition. Despite the absence of accurate details about the diet composition, it is tempting to hypothesize that the identified microbial profiles could reflect the lifestyles of different individuals. In fact, individuals originating from Africa and South America, who tend to have a diet characterized more by local and traditional products and less influenced by globalization, seemed to show a microbiota more similar to non-urbanized populations characterized by a high abundance of species belonging to the *Prevotella* and *Treponema* genera (53–56).

Conversely, multivariable analysis, based on the EC class composition of the key enzymes involved in the human diet (Fig. 4; Table S5), showed that the individuals from South America, Africa, and Asia possessed the highest number of negative correlations, i.e., 18, 11, and 11, respectively, compared with Europe, suggesting broader functional diversity or different ecological adaptations in the microbiome of these populations (53, 57). Furthermore, the observed negative correlations remained consistent across different age groups, suggesting a stable pattern in microbiome functionality over time (Fig. 4). These results could corroborate the notion that lifestyle substantially influences the intestinal microbiota, which, in turn, can exert a variable impact on the host, generally maintaining stability in terms of composition and functionality throughout human life. These preliminary results need further investigation using more accurate metagenomic data sets with detailed metadata on lifestyle factors, such as diet composition, use of drugs/antibiotics, and medical treatments. A deeper understanding of these aspects will enable us to better interpret lifestyle impact on the microbiome and its variation across different populations across the human life span.

## DISCUSSION

The human intestinal microbiota is widely recognized to play a key role in human health, so it has been the focus of extensive scientific research. However, knowledge regarding the evolution of gut microbiota over an individual’s lifetime has been limited. In this context, we decided to perform an extensive pooled analysis based on public and new shotgun metagenomic data sets of the human gut microbiota throughout the human life span, elucidating the dynamic nature of this complex ecosystem. The pooled analysis, encompassing a total of 6,653 fecal samples, identified an increase in microbial diversity in early life, followed by relative stability during adolescence, highlighting the continuous interplay between the microbiome and the host aging. Moreover, the statistical power of this pooled analysis allowed the identification of a potential age-related core microbiota at the bacterial species level. In detail, the samples representing the early stages of life showed the simplest core microbiota composition, mainly represented by *B. longum* and *E. coli* species. In contrast, adolescent and adult samples showed a more extensive and diversified core microbiota, supporting the hypothesis that the gut microbiota displays considerable changes in composition during the initial stages of development and then shifts to taxonomic stability with advancing age. Intriguingly, taxa, initially classified as accessories in the early stages of life, often become core components in subsequent stages. This transition could highlight a dynamic reorganization within the microbiota, characterized by shifting relative abundances rather than just the progressive acquisition and loss of taxa.

Moreover, the shotgun metagenomic approach allowed to investigate the functional capabilities of the gut microbiome. Specifically, the metagenomic analysis unveiled functional variations linked to age, particularly in the metabolism of carbohydrates and fibers, probably indicating a co-evolution of the microbiome and host influenced by dietary factors. Additionally, the functional analysis revealed possible associations with the biosynthesis of B vitamins and, in particular, with thiamine and niacin metabolisms during early life, suggesting a potential role of the microbiota in shaping human physiology, such as the functions of nervous and immune systems (58, 59).

Furthermore, an exploratory preliminary multivariable analysis investigated the relationship between the human gut microbiome and the geographical origin of the individual. The analysis revealed possible differences in microbiota profiles between continents. In detail, the gut microbiomes from South America and Africa showed distinct microbial compositions compared with other continents, probably related to more traditional diets that are less influenced by globalization. These trends persisted across age groups, indicating temporal microbiota stability. Similarly, the analysis of specific EC classes suggested a possible functional diversity related to geographical origin.

Such findings highlighted the co-evolution of the gut microbiota with the host throughout the human life span, revealing a bacterial adaptation to the host’s habits and its potential influence on host physiology. Nevertheless, the uneven distribution of samples across different groups of age and the lack of detailed metadata concerning, among others, diet composition and lifestyle could represent possible limitations of this study that should be overcome through more complex metagenomic analyses, allowing a more comprehensive understanding of the intricate interplay between gut microbiota and human health across the life span. Furthermore, while the current approaches based on genomic databases used for classifying bacterial populations often lack precise species-level identification, they remain a critical tool for microbial analysis. However, the ongoing expansion and enhancement of these genomic databases are expected to significantly improve the accuracy of microbial species identification of the human gut microbiota. Such advancements are crucial for deepening the understanding of the gut microbiota’s role in human health across various life stages. Despite these limitations, our study’s approach is considered effective for obtaining a comprehensive bacterial profile, particularly compared to other methods based on marker genes (42, 60).

## MATERIALS AND METHODS

### Selection and collection of samples included in the pooled analysis

In this pooled analysis-based study, we retrieved 79 publicly available data sets from studies regarding the human gut microbiome for a total of 6,186 samples from 37 different nations (Table S1). In particular, we selected shotgun metagenomic data sets obtained by an Illumina sequencing platform to avoid the input data’s variability as much as possible. In addition, we included 467 Italian adult healthy individuals collected as part of the Parma Microbiota project (Comitato Etico dell'Area Vasta Emilia Nord, Emilia-Romagna Region, Italy, under the ID 1107/2020/TESS/UNIPR) (Table S1). These Italian fecal samples, once collected, were immediately inactivated with DNA/RNA shield buffer (Zymo Research, USA) and subsequently delivered to the Laboratory of Probiogenomics of Parma University, where the analysis of bacterial DNA libraries by shotgun metagenomic and the bioinformatic analysis of raw sequencing data were performed.

### Shallow shotgun sequencing

According to the manufacturer’s instructions, DNA library preparation was performed using the Nextera XT DNA Sample Preparation Kit (Illumina, San Diego, CA, USA). First, 1-ng input DNA from each sample was used for the library preparation, which underwent fragmentation, adapter ligation, and amplification. Then, Illumina libraries were pooled equimolarly, denatured, and diluted to a concentration of 1.5 pM. Next, DNA sequencing was performed on a MiSeq instrument (Illumina) using a 2 ×  250-bp Output Sequencing Kit together with a deliberate spike-in of 1% PhiX control library.

### Taxonomic classification of sequence reads

Taxonomic profiling of sequenced and downloaded reads was performed employing the METAnnotatorX2 bioinformatic platform (42, 61). In detail, the fastq files were filtered to remove reads with the quality of <25 and to retain reads with a length of >100 bp. Subsequently, human host DNA filtering was performed through Bowtie 2 software (62, 63), following the METAnnotatorX2 manual (42). Afterward, the taxonomic classification of 100,000 reads was achieved by means of MegaBLAST (64) employing a manually curated and pre-processed database of genomes retrieved from the National Center for Biotechnology Information, following the METAnnotatorX2 manual (42).

### Functional prediction

Functional profiling of the sequenced reads was performed with the METAnnotatorX2 bioinformatic platform (42, 61). Functional classification of reads was performed to reveal metabolic pathways based on the MetaCyc database (release 24.1) (49) through RAPSearch2 software (65, 66).

### Statistical analysis

ORIGIN 2021 (https://www.originlab.com/2021) and SPSS software (www.ibm.com/software/it/analytics/spss/) were used to compute statistical analyses. In detail, pairwise Kruskal-Wallis test analyses tested differences in alpha diversity that is calculated through species richness and Shannon index. Moreover, the similarities between samples (beta-diversity) were calculated by the Bray-Curtis dissimilarity matrix based on species abundance, using the “vegdist” function (from vegan_2.5–7) on RStudio (http://www.rstudio.com/). The range of similarities is calculated between values 0 and 1. Beta-diversity was represented through PCoA using the function “ape” of the Rsuite package (67). Moreover, the available metadata and the various detected bacterial species were tested and plotted on the PCoA using the “envfit” and “plot” functions from vegan (version 2.5–7), respectively, through RStudios (http://www.rstudio.com/). PERMANOVA analyses were performed on RStudio using 999 permutations to estimate *P* values for population differences in PCoA analyses with adonis2 package (from vegan_2.5–7). Furthermore, a correlation analysis between the available metadata and the various detected bacterial species of all samples was performed through Spearman’s rank correlation coefficient using “rcorr” function (from Hmisc_4.6–0; https://CRAN.R-project.org/package=Hmisc), and only significant statistical results were retained. The false discovery rate (FDR) correction based on Benjamini and Hochberg correction (68) and calculated using RStudio through “p.adjust” function (from base package stats) was applied to statistically significant results. In detail, correlation analysis was performed between metabolic reactions revealed through the metagenomic analysis and the 104 bacterial species, which exhibited a significant relationship with the host’s age. Afterward, we focused our interest on the 46 bacterial taxa that showed significantly higher relative abundance in at least one of the age groups calculated through analysis of variance (ANOVA) test analysis and multiple comparison analyses Tukey’s HSD test and on the main key enzymes involved in the metabolism of the various components of the infant and/or adult human diet, such as human milk oligosaccharides, carbohydrates, and fibers, or in the metabolism of the main microbial products important for the host, such as B vitamins and short-chain fatty acids.

Furthermore, multivariable statistical analysis based on MaAsLin2 software (52) was performed to identify potential variations in the human gut microbiota based on geographical origin. In detail, the multivariable analysis allowed to investigate the possible correlation between the human gut microbiome and the geographical origin of the host. We focused on the continent of origin to reinforce the statistical power of the analysis. The analysis based on MaAsLin2 software was performed separately for each age group, considering the continent of origin, the microbiota composition, and the EC composition of the key enzymes involved in the human diet (see above). Moreover, European individuals were selected as analysis references, primarily due to the higher average number of samples across each age group. Afterward, we focused on the attention on the taxa that exhibit significant statistical correlations across all age groups in at least one continent group.

## Data Availability

Raw Italian shotgun metagenomic data sequences are accessible through SRA under study accession number PRJNA1046438.

## SUPPLEMENTAL MATERIAL

Figure S1 msystems.01294-23-s0001.tif Workflow of the pooled analysis performed and species richness identified by subjects of each age subgroup.

Legends msystems.01294-23-s0002.docx Supplemental material legends.

Table S1 msystems.01294-23-s0003.xlsx List of the public bioprojects included in the pooled analysis.

Table S2 msystems.01294-23-s0004.xlsx Metadata of the samples included in the pooled analysis.

Table S3 msystems.01294-23-s0005.xlsx PERMANOVA statistical analysis based on the Bray-Curtis dissimilarity matrix calculated the inter-individual differences between age groups.

Table S4 msystems.01294-23-s0006.xlsx Core and accessory microbiota calculated on the subjects included in the pooled analysis.

Table S5 msystems.01294-23-s0007.xlsx Correlation analysis between the bacterial species and enzymatic reaction identified in pooled analysis.

Table S6 msystems.01294-23-s0008.xlsx Multivariate analysis through MaAslin2 software based on bacterial species, age groups, and geographical origin.

ASM does not own the copyrights to Supplemental Material that may be linked to, or accessed through, an article. The authors have granted ASM a non-exclusive, world-wide license to publish the Supplemental Material files. Please contact the corresponding author directly for reuse.
