# Supplementary figures and images for: Taxonomic and metabolic development of the human gut microbiome across life stages: a worldwide metagenomic investigation
Source: mSystems. 2024 Mar 5;9(4):e01294-23. doi: 10.1128/msystems.01294-23 (PMC11019788; doi:10.1128/msystems.01294-23)

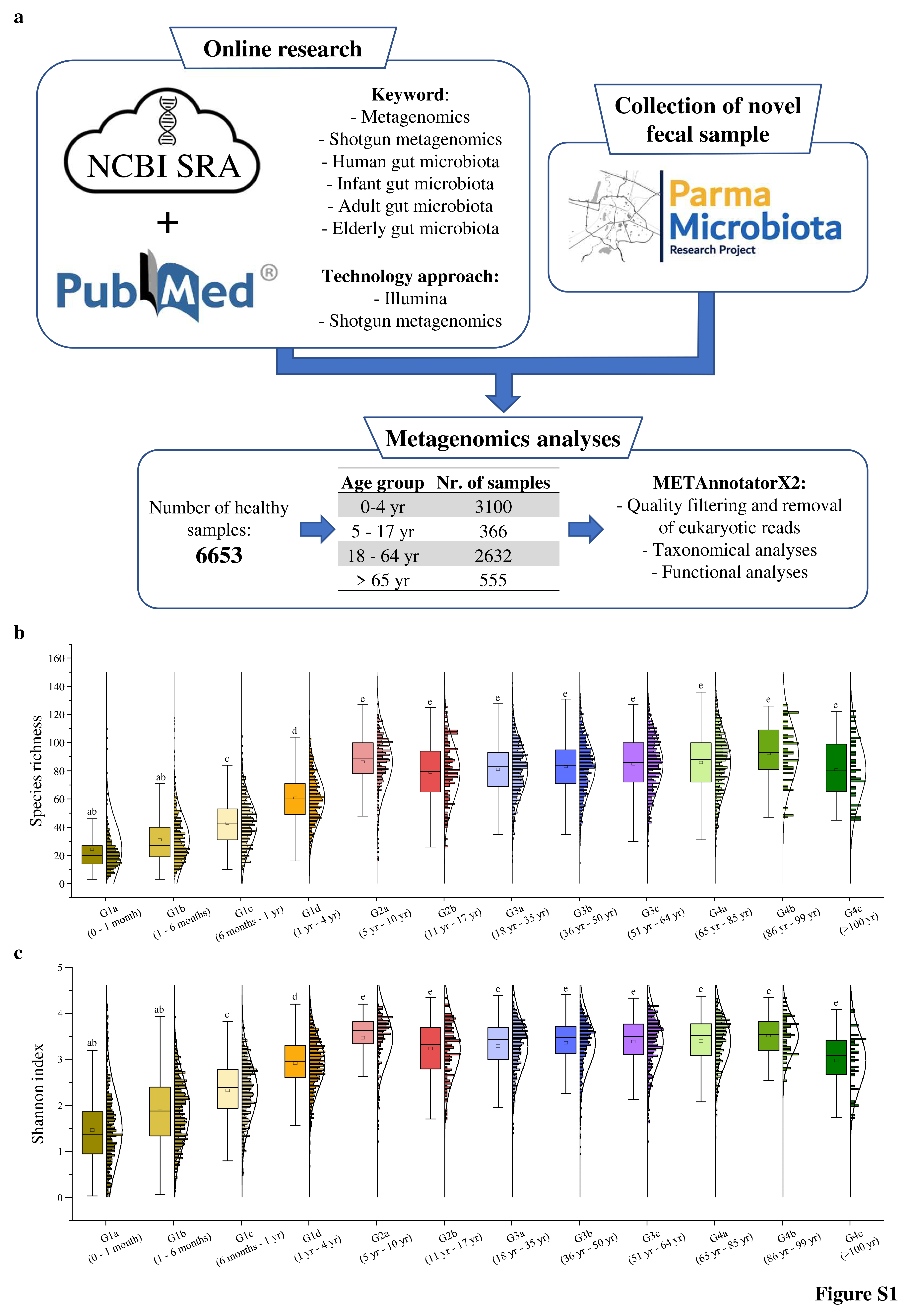

Supplement: Figure S1 — Workflow of the pooled analysis performed and species richness identified by subjects of each age subgroup. [file msystems.01294-23-s0001.tif]
