# Supplementary material for: Taxonomic and metabolic development of the human gut microbiome across life stages: a worldwide metagenomic investigation
Source: mSystems. 2024 Mar 5;9(4):e01294-23. doi: 10.1128/msystems.01294-23 (PMC11019788; doi:10.1128/msystems.01294-23)
Supplement: Legends — Supplemental material legends. [file msystems.01294-23-s0002.docx]

**Taxonomic and metabolic development of the human gut microbiome across life stages: a worldwide metagenomic investigation**

Leonardo Mancabelli^1,2^, Christian Milani^2,3^, Rosita De Biase^3^, Fabiana Bocchio^3^, Federico Fontana^3^, Gabriele Andrea Lugli^3^, Giulia Alessandri^3^, Chiara Tarracchini^3^, Alice Viappiani^4^, Flora De Conto^1^, Antonio Nouvenne^1,2,4^, Andrea Ticinesi^1,2,4^, Ovidio Bussolati^1,2^, Tiziana Meschi^1,2,4^, Rossana Cecchi^1,2^, Francesca Turroni^2,3^, Marco Ventura^2,3^

**Supplementary materials legends**

**Figure S1**: Panel (a) reports the workflow of the pooled analysis performed. Panel (b) displays the Whiskers plot representing the species richness identified by subjects of each age subgroup. The x‐axis represents the different age groups, while the y‐axis indicates the number of species. The 25th and 75th percentiles determine the boxes. The whiskers are determined by the 1.5 interquartile range (IQR). The line in the boxes represents the median, while the square represents the average. Different lowercase letters indicate significant differences at p‐value < 0.05 calculated through pairwise Kruskal-Wallis Test analyses. In detail, groups with the same letter are not significantly different from each other, while groups with different letters are considered statistically distinct. Panel (c) reports the Whiskers plot representing the alpha diversity calculated through the Shannon index identified by subjects of each age group. The x‐axis represents the different age groups, while the y‐axis indicates the Shannon index. The 25th and 75th percentiles determine the boxes. The whiskers are determined by the 1.5 interquartile range (IQR). The line in the boxes represents the median, while the square represents the average. Different lowercase letters indicate significant differences at p‐value < 0.05 calculated through pairwise Kruskal-Wallis Test analyses. In detail, groups with the same letter are not significantly different from each other, while groups with different letters are considered statistically distinct.

**Table S1**: List of the public bioprojects included in the pooled analysis.

**Table S2:** Metadata of the samples included in the pooled analysis.

**Table S3**: PERMANOVA statistical analysis based on the Bray-Curtis dissimilarity matrix calculated the inter-individual differences between age groups.

**Table S4**: Core and accessory microbiota calculated on the subjects included in the pooled analysis.

**Table S5**: Correlation analysis between the bacterial species and enzymatic reaction identified in pooled analysis.

**Table S6**: Multivariate analysis through MaAsLin2 software based on bacterial species, age groups and geographical origin.
